# Supplementary material for: Expanding mental health services in low- and middle-income countries: A task-shifting framework for delivery of comprehensive, collaborative, and community-based care
Source: Glob Ment Health (Camb). 2023 Feb 27;10:e16. doi: 10.1017/gmh.2023.5 (PMC10579648; doi:10.1017/gmh.2023.5)
Supplement: Supplementary file 1 [file S2054425123000055sup001.docx]

**APPENDIX A**

**BACKGROUND: EXISTING MODELS, FRAMEWORKS AND GUIDANCE**

The *Comprehensive, Collaborative Community-based Care (C4)* *Framework* for LMICs builds upon existing models, particularly the *World Mental Health Report: Transforming Mental Health for All* (World Health Organization, 2022), *WHO Service Organization Pyramid for an Optimal Mix of Services for Mental Health* (World Health Organization, 2009*b*)*,* the Inter-Agency Standing Committee (IASC) Guidelines for Mental Health and Psychosocial Support in Emergencies (which forms the basis for programming by most humanitarian and many development organizations), and the *International Medical Corps (IMC) Stepped Care Levels of Integrated Mental Health and Psychosocial Support Services and Protection Services* (International Medical Corps, 2017*b*) models for mental health services delivery. It also reflects key recommendations from the recent *Lancet Commission on Global Mental Health and Sustainable Development* (Patel et al., 2018), Thornicroft and Tansella’s *Balanced Care Model for Global Mental Health* (Thornicroft et al., 2013), and on an unpublished model proposed by Derrick Silove. These models are briefly described below.

**World Mental Health Report: Transforming Mental Health for All**

The primary aim of this report was to inspire and inform the transformation required to ensure better mental health for all – by deepening the value and commitment to mental health; re-shaping the environments that influence mental health; and by strengthening community-based services (World Health Organization, 2022). It highlighted the need to address key factors which prevent individuals from seeking help for mental health conditions, including the poor quality of services, low levels of health literacy in mental health, and stigma and discrimination. The key areas for action identified included: governance and leadership; finance; public awareness; and competencies for mental health care. Promotion and prevention at all stages of life are stressed to enhance mental well-being and resilience, prevent the onset and impact of mental conditions, and decrease the need for mental health care. For mental health services delivery, the report proposed transforming mental health by strengthening community-based care for all in need through a major reorganization of mental health services to shift the locus of care for severe mental health conditions away from psychiatric hospital towards community-based mental health services and to scale up care for common conditions such as depression and anxiety.

In outlining the major restructuring and scaling up required to transform mental health services to meet the needs of all, a multi-sectoral, community-based mental health care system is emphasized – stressing the importance of providing person-centered, human-rights-based and recovery-oriented care. Under this proposed approach, social and informal support delivered by community providers (e.g., community workers, peers) complement formal services. The *Transforming Mental Health for All* model is comprised of a network of interconnected services that include:

1) *Mental Health Services Integrated in General Health Care* through task-sharing with non-specialist health care providers or adding dedicated mental health staff in primary care settings, general hospitals, and disease- or population-specific health programs such as those focusing on HIV/AIDS, refugees, or maternal and child health.

2) *Community Mental Health Services* that focus on meeting the needs of people living with mental health conditions and their families, which may include community mental health centers or multidisciplinary teams as well as peer support services, crisis services, psychosocial rehabilitation programs and, where feasible, supported living through small-scale residential facilities.

3) *Services Beyond the Health Sector* that deliver mental health care – including psychosocial supports provided by a diverse range of community providers -- in non-health settings such as schools, workplaces and criminal justice settings and support access to key social services, including housing, child protection, education, employment, social benefits, and livelihood support. Complementing health interventions with these key social services is described as critical to achieve recovery goals, and supported living services essential to promote deinstitutionalization.

Central to the success of this model and the expansion of the specialist workforce is achieving and maintaining competencies in mental health by care providers at all levels, ranging from individuals and community providers to general and specialist health care workers. With this approach, a broad range of providers who are not mental health care specialists, including community workers, lay volunteers, teachers, police officers and justice system staff, are engaged to develop specific skills to deliver basic mental health interventions.

To expand the specialist workforce and address the vast care gap for common mental health conditions such as depression and anxiety, scaling up use of non-specialist psychological counseling or digital self help is proposed. A key strategy to accomplish this is through the use of psychological counseling programs that recruit, train and deploy non-specialist counselors to deliver group or individual evidence-based psychological interventions. These non-specialist counselors for depression and anxiety can include a wide range of people from community workers, volunteers or peers with as little as ten years of education as well as individuals with university degrees but without specialist mental health training.

In addition to evidence-based psychological interventions provided by trained non-specialist counselors, social and informal supports delivered by community providers with mental health care competencies are employed in this model to complement formal services and are key to mental health prevention, promotion and ensuring enabling environments. These social and informal supports could be provided by individuals with lived experience, lay volunteers, community workers, coordinators of user groups, religious counselors, teachers, hairdressers, lawyers, police officials, justice system guards or family and friends. The development of competencies for self-care is also stressed.

The *Transforming Mental Health for All* person-centered, recovery-based approach was proposed to help ensure all people have access to a range of services and supports, from promotion and prevention to treatment and rehabilitation. Under this multi-sectoral model, care is to be coordinated across different levels and sites within and beyond the health sector, according to peoples’ needs throughout the life-course (World Health Organization, 2016).

**WHO Services Organization Pyramid for Optimal Mental Health Care**

The 2003 *WHO Services Organization Pyramid* provides a foundational set of concepts for mental health system development, recommending that countries prioritize: 1) Promoting self-care; 2) Building informal community care services; 3) Integrating mental health services into primary health care to facilitate identification and intervention in primary care settings and referrals to specialty care; 4) Building community mental health services to provide mental health care in the community; 5) Developing mental health services in general hospitals; and 6) Limiting the use of psychiatric hospitals (World Health Organization, 2009*b*). A primary focus is on treating mental health conditions as early as possible, holistically and close to the person’s home to achieve the best outcomes. Most care is to be provided informally by facilitated, trained, and mentored family and community networks, self-care, and through peer support (World Health Organization, 2009*d*).

The WHO model emphasizes complementing primary care with referrals, support, supervision and linking to informal and community-based mental health services (World Health Organization, 2009*a*). Training community workers and health care providers and linking them to primary, secondary and tertiary health care services in scalable psychological interventions is key to facilitating the availability of mental health services (World Health Organization, 2009*e*). Support, supervision, collaboration, information-sharing and education across the levels of care are considered essential (World Health Organization, 2009*b*).

Self-care is the first level and foundation of the WHO model. Involving individuals in their own mental health care promotes recovery and mental health and wellbeing since many individuals manage their mental health conditions by themselves or with family and friends’ support rather than the formal health care system. Informal community care services are the second level of care. Care providers include stakeholders in social welfare, protection, education, judiciary or civil society providing formal or informal support for mental health, faith-based organizations and traditional healers. Interventions focus on awareness and mental health literacy; prevention and promotion; referral, management and rehabilitation (World Health Organization, 2009*c*). Management activities can include provision of psychological therapy, psychoeducation, stress management, vocational support and consumer self-help groups. Mapping community resources and stakeholders that could provide formal or informal support for mental health and identifying and strengthening these referrals and care pathways is emphasized. Community health workers and other care workers are encouraged to manage cases in order to better coordinate treatment and care in the community (World Health Organization, 2018).

Primary care mental health services are the third level. Services include mental health promotion and prevention, early identification and intervention, psychiatric management (including management of acute and chronic health care needs), counselling for common mental health conditions and referrals to other levels. Services at the primary health care level are considered the most accessible, affordable and acceptable for communities. Specialized community mental health services (e.g., day centers, rehabilitation services, mobile crisis teams, therapeutic and residential supervised services, group homes, home help, assistance to families and other support services such as school-based psychological services) and psychiatric services in general hospitals form the fourth level of care in this model. Mental health professionals responsible for mental health services include psychiatrists, community psychiatric nurses, psychologists, psychiatric social workers, occupational therapists and community psychiatric workers. Finally, long-stay facilities and specialist psychiatric services are the fifth level of care for the few who are severely ill, intervention-resistant or have complex presentations requiring specialized management.

**Inter-Agency Standing Committee (IASC) Guidelines for Mental Health and Psychosocial Support in Emergencies**

The *Inter-Agency Standing Committee (IASC) Intervention Pyramid for MHPSS in Emergencies* was designed to help humanitarian actors and communities plan, establish and coordinate a set of minimum multi-sectoral, inter-agency responses to protect and improve people’s mental health and psychosocial well-being during an emergency (Inter-Agency Standing Committee, 2007). It seeks to build local capacities, support self-help and strengthen the resources already present through a layered system of complementary supports to meet the needs of different groups (Inter-Agency Standing Committee, n.d.). The model emphasizes integrating activities into wider systems, including existing community support mechanisms, formal and non-formal school systems, general health and mental health services, social services, etc., to reach people sustainably while minimizing stigma.

The first layer of the IASC model refers to ‘social considerations in basic services and security’ which refers to how basic services can be established that best promote psychosocial well-being. Community and family support form the second layer for individuals who are able to maintain their mental health and psychosocial well-being if they receive help in accessing key community and family supports. Layer three includes focused, non-specialized support for individuals who require more focused individual, family or group interventions by trained or supervised workers who do not necessarily have years of specialized mental health or psychosocial services training. This layer includes basic psychosocial and mental health care by primary health care workers. The fourth layer consists of specialized services for individuals whose suffering, despite the supports already described, is intolerable and may have significant functional impairments. Level four services include specialist psychological or psychiatric support.

**International Medical Corps (IMC) Stepped Care Levels of Integrated MHPSS Services**

The Non-Governmental Organization (NGO) International Medical Corps (IMC) developed an MHPSS services framework for stepped mental health care for emergent and non-emergent situations (International Medical Corps, 2017*b*). This framework is integrated into comprehensive mental health case management services, with an interdisciplinary team working collaboratively to address individuals’ holistic needs. Individuals are managed at the lowest level of care possible, moving up or down levels of care depending on their needs. These levels of care are:

- ***Level one: community interventions targeting the entire population.*** Provided by community-level health and social services workers tasked with case finding, provision of basic psychosocial support, follow up, community education, and mapping and linking with existing services and referral pathways. Workers also monitor individuals’ progress and advise on appropriate care levels in consultation with technical specialists on the care team.
- ***Level two: core psychosocial interventions targeting individuals with psychological distress or mental health problems that impair functioning.*** Provided by psychosocial workers and case managers who conduct assessments (biopsychosocial and risk), develop and implement care plans, implement safety plans with the care team, conduct support and psychoeducation groups and scalable psychological interventions, provide follow-up and discharge.
- ***Level three: pharmacologic and basic non-pharmacologic interventions,*** ***targeting individuals with mental health problems in need of medications and those with severe mental health issues, including self-harm.*** Provided by general practitioners trained and supervised in mental health. They conduct mental health assessments; prescribe and manage medications; provide psychoeducation and coordinate care with psychosocial workers.
- ***Level four: psychiatric care and emergency interventions*** ***targeting those with mental health problems that are severe or complex*** and do not improve. Key functions include brief psychiatric assessments, medication management and psychoeducation regarding side effects. These functions are intended to be provided by a psychiatrist.

The IMC approach seeks to optimize individuals’ functioning by providing services that meet multiple needs (International Medical Corps, 2017*a*). Services are client-centered, empowerment-oriented and based on protection of human rights. The multidisciplinary intervention team may consist of: psychosocial workers and case managers; psychologists or psychiatrists (where available); mhGAP-trained healthcare workers; mental health nurses; and pharmacists. The team works together to ensure a coordinated approach across the levels of care. Ideally, multi-disciplinary team meetings are held regularly to discuss complex and high-risk cases and coordinate management. The model emphasizes case consultations for particularly complex and high-risk cases given the many challenges facing management teams (including protection-related concerns and ethical dilemmas) to ensure appropriate, principled and coordinated interventions. The holistic approach to understanding needs (e.g., safety of self, safety of children, safety at home) is important. Additional benefits of case consultations include more nuanced technical support and quality oversight of services. Designated MHPSS providers take the lead in ensuring clear, understood protocols related to clinical consultations for the multidisciplinary team.

**Lancet Commission on Global Mental Health and Sustainable Development “Balanced Care Model”**

The *Lancet Commission on Global Mental Health and Sustainable Development*’s “Balanced Care Model” balances focus on intervention, rehabilitation care and recovery with equal emphasis on promotion of mental health and prevention of mental health conditions, with particular attention to early interventions (Patel et al., 2018). It uses a staging approach with intervention at all stages from wellbeing onwards. This entails addressing early common mental distress symptoms such as anxiety or low mood with evidence-based community and intersectoral interventions provided outside the health care system, such as employment opportunities, child protection services, long-term social care and suicide prevention.

The model proposes scaling up: 1) Task-shifting of psychosocial interventions to non-specialized workers as the foundation of the mental health care system; 2) Coordination between these workers with primary and specialist care; 3) Digital platforms to deliver interventions; and 4) Community-based interventions to enhance demand for care. The first step is self-delivered interventions for mild to moderate conditions, with online and mobile platforms proposed. The second step includes psychosocial interventions for more severe conditions in routine care settings or homes by community health workers or lay counsellors. The final step is specialist or physician consultation for very severe cases, including pharmacologic interventions, more complex psychotherapies, and other physical health therapies.

**Partners In Health (PIH) Value Chain and Planning Matrix Approach to Building Mental Health Systems of Care**

Applying guidance from WHO and the other resources described above to existing front-line providers within government systems, across district hospitals, primary care clinics and communities, the NGO Partners In Health (PIH) focuses on the challenges of providing comprehensive services in difficult circumstances (Raviola et al., 2019; Partners In Health, 2019). PIH has sought to specify the flow of service users through emerging, integrated health care delivery systems that bridge psychiatric, psychological and psychosocial services with social support. This has led to the *value chain* framework, embedded within a *service delivery planning matrix*, which together apply a systems-level analysis to the complex processes and interventions necessary across all health system levels to deliver high-value care for service users (Kim et al., 2013; Partners in Health, n.d.).

A *value chain* describes the system elements essential for quality care delivery to achieve optimal health outcomes from when a service user enters the system to when they leave. The system extends from hospitals to primary care centers to communities and households. The PIH mental health value chain encompasses an interdependent core set of discrete care activities across health facility and community levels, along with their sequence and organization, which was defined through the experience of local implementing teams in collaboration with Ministries of Health over a decade of successful clinical service implementation, and through subsequent theory of change processes across Rwanda, Haiti, Chiapas (Mexico), Liberia and elsewhere (Coleman et al., 2021). Individual activities across the value chain should be considered in relation to other activities in order to maximize value for patients (Rhatigan et al., 2009). The core bundles of care activities within the value chain include: Crisis Response; Prevention; Case-Finding; Assessment; Treatment; Follow-up; and Reintegration.

**Crisis response*.*** Crisis response includes use of international guidance such as the Inter Agency Standing Committee Guidelines (Inter-Agency Standing Committee, 2007) and other best practices for needs assessment, preparedness and response efforts, including Psychological First Aid and other individual and community-focused interventions, to address ongoing and emerging local and global crises. The imperative to take advantage of humanitarian crises as opportunities to “Build Back Better,” strengthening government-run community-based and primary-care systems over the long term, is implicit in the inclusion of crisis response capacity as a key component of the value chain.

**Prevention.** Prevention seeks to reduce the incidence of, and improve trajectories for, mental health conditions. Prevention activities are performed across all service levels, with a particular focus on the community, and include anti-stigma campaigns, educational programs, school-based programs, and anti-poverty and other initiatives designed to reduce social and economic contributors to poor mental health.

**Case-finding.** Case-finding identifies those with possible or probable mental health conditions and provides an entry point to care. Case-finding occurs in the community as well as primary care services, and includes proactive case-identification and detection, formal screening, and referral management.

**Assessment.** Assessment occurs primarily at facilities including primary care centers and specialized mental health facilities and includes clinical and biopsychosocial assessments, including medical evaluation, diagnosis, enrollment in care, and care planning across communities and facilities.

**Treatment.** Treatment is wide-ranging and occurs in both primary and secondary care facilities, and communities at the individual and group level. Treatment encompasses psychoeducation, psychosocial interventions including psychosocial rehabilitation, psychotherapies, safety planning and emergency interventions, medication management, and inpatient hospitalization.

**Follow-up.** Follow-up includes ongoing monitoring of clinical progress and functioning, treatment adherence support, case management, care coordination, and referral and counter-referral among service levels.

**Reintegration.** Reintegration seeks to optimize quality of life and full community integration for people living with mental health conditions. Activities occur in health, social services and other sectors, and include activities to promote individual and family functioning such as psychoeducation, peer support, caretaker support, vocational training, social assistance, anti-stigma campaigns, and stakeholder engagement with communities, local authorities, and government.

The mental health care delivery value chain is embedded within a *service delivery planning matrix* that includes strategies for service delivery platform elaboration, including skills mapping for human resource management, implementation, and spread and sustainability. The PIH value chain and service delivery planning matrix have facilitated the delivery of over 264,000 facility- and remote-based (related to COVID-19) mental health visits from 2016 to 2022 across 105 public sector health care facilities. This has included enrolling over 62,000 people into mental health care for the first time, as well as a 6-fold increase in patients treated annually from 2016 to 2020. The value chain elements provide a blueprint from which system builders and implementers can plan service delivery activities, training, monitoring and evaluation, and quality improvement initiatives. It informs experience-based systems design and elaboration of functional management structures, increasing use of operational best practices and lessons to be disseminated among those responsible for operating mental health and psychosocial care systems, including in emergencies. Integrating emergency response as outlined by IASC and basic non-specialist practices and as outlined by WHO mhGAP within such implementation-focused frameworks, the value chain and matrix facilitate nimble and comprehensive systemic responses to new crises such as COVID-19 in a rapidly changing world (Smith and Raviola, 2020).

**APPENDIX A REFERENCES**

Coleman, SF, Mukasakindi, H, Rose, AL, Galea, JT, Nyirandagijimana, B, Hakizimana, J, Bienvenue, R, Kundu, P, Uwimana, E, Uwamwezi, A and Contreras, C (2021) Adapting problem management plus for implementation: lessons learned from public sector settings across Rwanda, Peru, Mexico and Malawi. Intervention 19(1), 58.

Inter-Agency Standing Committee, 2007. IASC guidelines on mental health and psychosocial support in emergency settings. Available at: https://www.who.int/publications/i/item/iasc-guidelines-for-mental-health-and-psychosocial-support-in-emergency-settings (accessed 15 February 2022).

Inter-Agency Standing Committee, n.d. Reference group for mental health and psychosocial support in emergency settings. Available at: https://interagencystandingcommittee.org/iasc-reference-group-on-mental-health-and-psychosocial-support-in-emergency-settings (accessed 15 February 2022).

International Medical Corps, 2017*a*. Approach to Mental Health and Psychosocial Support Case Management. 2017*a*. International Medical Corps. Available at: https://cdn1.internationalmedicalcorps.org/wp-content/uploads/2017/07/International-Medical-Corps-2012-Our-Approach-to-Mental-Health-Psychosocial-Support.pdf (accessed 15 February 2022).

International Medical Corps, 2017*b*. MHPSS Services Framework. Available at: https://www.mhinnovation.net/sites/default/files/downloads/resource/IMC%202017%20MHPSS%20services%20framework.docx (accessed 15 February 2022).

Kim, JY, Farmer, P and Porter, ME (2013) Redefining global health-care delivery. The Lancet 382(9897), 1060-1069.

Partners in Health, 2019. Mental Health at Partners In Health (PIH). Available at: https://storymaps.arcgis.com/stories/8dca051575aa4dd983e9fe1e21bcff6b (accessed 15 February 2022).

Partners In Health, n.d. Mental Health Service Planning Matrix. Partners In Health. Available at: https://www.pih.org/MHMatrix (accessed 15 February 15, 2022).

Patel, V, Saxena, S, Lund, C, Thornicroft, G, Baingana, F, Bolton, P, Chisholm, D, Collins, PY, Cooper, JL, Eaton, J and Herrman, H (2018) The Lancet Commission on global mental health and sustainable development. The Lancet 392(10157), 1553-1598.

Raviola, G, Rose, A, Fils-Aimé, JR, Thérosmé, T, Affricot, E, Valentin, C, Daimyo, S, Coleman, S, Dubuisson, W, Wilson, J and Verdeli, H (2020) Development of a comprehensive, sustained community mental health system in post-earthquake Haiti, 2010–2019. Global Mental Health 7, e6.

Rhatigan, J, Jain, SH, Mukherjee, J and Porter, ME (2009) Applying the care delivery value chain: HIV/AIDS care in resource poor settings. Harvard Business School.

Smith, SL and Raviola, GJ (2020) ‘Jack be nimble, Jack be quick…’: mental health and psychosocial response in the time of coronavirus. Global Mental Health 7, e21.

Thornicroft, G and Tansella, M (2013) The balanced care model: the case for both hospital-and community-based mental healthcare. The British Journal of Psychiatry 202(4), 246-248.

World Health Organization, 2009*a.*  Improving Health Systems and Services for Mental Health, pp. 1. Available at: https://www.who.int/mental_health/policy/services/mhsystems/en/ (accessed 15 February 2022).

World Health Organization, 2009*b*. Improving Health Systems and Services for Mental Health, pp. 21. Available at: https://www.who.int/mental_health/policy/services/mhsystems/en/ (accessed 15 February 2022).

World Health Organization, 2009*c*. Improving Health Systems and Services for Mental Health, pp. 22-3. Available at: https://www.who.int/mental_health/policy/services/mhsystems/en/ (accessed 15 February 2022).

World Health Organization, 2009*d*. Improving Health Systems and Services for Mental Health, pp. 39. Available at: https://www.who.int/mental_health/policy/services/mhsystems/en/ (accessed 15 February 2022).

World Health Organization, 2009*e*. Improving Health Systems and Services for Mental Health, pp. 40. Available at: https://www.who.int/mental_health/policy/services/mhsystems/en/ (accessed 15 February 2022).

World Health Organization, 2016. Framework on integrated, people-centered health services. Sixty-ninth World Health Assembly, Geneva, 15 April 2016. Provisional agenda item 16.1. Available at: https:// apps.who.int/gb/ebwha/pdf_files/WHA69/A69_39-en.pdf?ua=1 (accessed 29 March 2022).

World Health Organization, 2018. mhGAP operations manual: mental health Gap Action Programme (mhGAP), pp.45-46. Available at: https://www.who.int/publications/i/item/mhgap-operations-manual (accessed 15 February 2022).

World Health Organization, 2022‎. World mental health report: transforming mental health for all. Available at: https://www.who.int/publications/i/item/9789240049338 (accessed 15 February 2022).
